# Body, gender, race and fatness in social studies. Gap and future

**DOI:** 10.3389/fsoc.2025.1525944

**Published:** 2025-05-09

**Authors:** Ahmed Nasser Alwulaii

**Affiliations:** Social Studies Department, College of Humanities and Social Sciences, King Saud University, Riyadh, Saudi Arabia

**Keywords:** body, gender, race, social studies, future

## Abstract

Sociologists have increasingly turned to the body as a critical aspect of both individual identity and social organization. Some scholars, such as, examine the body's actions within social relations, while others, such as, have demonstrated the power of discourse in controlling and shaping bodies. Additionally, other scholars, like, view the body as a form of physical capital and argue that bodies reflect social class through three major interrelated factors: social location, taste, and habitus. However, sociological studies on the body lack certain dimensions that need to be addressed. In the next section, I will briefly illustrate some of the key gaps in sociological studies of the body concerning gender, race, and fatness. These gaps include an overemphasis on women's bodies, a predominant focus on the white race, a concentration on Western societies while neglecting other parts of the world, and a failure to consider fatness as a social issue. Furthermore, I will briefly discuss future perspectives on sociological studies regarding the intersections of body, gender, and fatness.

## Introduction

In the past few years, there has been a significant increase in academic attention to the body (Shilling, 2012). This growing interest in body studies within sociology reflects how the body has become a central topic in sociological research (Shilling, 2012). Social studies on the body have extensively explored how individuals engage with their bodies in various contexts (Shilling, 2007). In other words, they have examined how race, class, gender, and fatness have shaped individuals' experiences with their bodies from the 1980s to the present. Sociologists have increasingly turned to the body as a critical aspect of both individual identity and social organization (Grogan, 1999). Some scholars, such as Goffman (1959), examine how the body operates within social interactions, while others, like Foucault (1980), have demonstrated the power of discourse in disciplining and regulating bodies. Yet other scholars see the body as a form of physical capital and argue that bodies bear social class because of three major interrelated factors: social location, taste, and habitus (Bourdieu, 1984). However, sociological studies on the body from different perspectives are missing key dimensions that need to be addressed. In the next section, I will briefly illustrate some of the issues and gaps in sociological studies of the body concerning gender, race, and fatness. Most sociological studies focus primarily on women's bodies, with a predominant emphasis on the white race. Additionally, research tends to center on Western societies while neglecting other parts of the world. Furthermore, fatness is not adequately considered as a social issue, as many studies approach it solely from a medical or psychological perspective rather than examining its social and cultural implications. In addition, I will briefly illustrate the perspective of future about the sociological studies in terms of body, gender, and fatness.

### Most social studies focusing on women's body/bodies

Researchers in sociology and other academic fields related to fatness and gender have argued that men are less concerned about their weight or body image compared to women (Wolf, 2013; Faludi, 2009; Bordo, 1993), Most researchers in different academic disciplines focus on women's bodies and body image. In the last century, women have been subjected to certain 'aesthetic pressures' more than men (Bell and McNaughton, 2007). Since the 1960s, scholarship has focused on women's bodies more than men (Gilman, 2004; Bell and McNaughton, 2007). However, some scholars have argued that men's current concern about their bodies is a new phenomenon (Bordo, 1993). Therefore, studies on the male body and men's body concerns have emerged only recently (Wolf, 2013; Faludi, 2009; Bordo, 1993).

This transformation is particularly noticeable in contemporary Western societies, where men increasingly dedicate substantial time and effort to enhancing their physical appearance (Bergling, 2007; Drummond, 2005; Duncan, 2010). Scholars and media outlets suggest that this trend stems from the growing emphasis on physical attractiveness within male culture, which prioritizes body aesthetics and appearance (Bergling, 2007; Drummond, 2005; Duncan, 2010, 2007; Kassel and Franko, 2000; Yelland and Tiggemann, 2003). The heightened focus on maintaining an ideal physique has been linked to rising concerns about body image among men, potentially increasing their susceptibility to body dissatisfaction and related psychological challenges (Boisvert and Harrell, 2010; Chaney, 2008; Morrison et al., 2004).

In short, studies on bodies and gender have generally focused more on women's experiences with their bodies than on men's experiences. Even though attention to studies on men's bodies has been increasing in recent years, this field still needs further expansion. For example, these studies give more attention to the negative consequences of not adhering to ideal cultural body standards for women, such as eating disorders, social anxiety, dissatisfaction, and depression, while paying little attention to men's bodies and the consequences of not meeting these ideal standards. As a result, this has created a gap in body-related studies between men and women. On the other hand, Middle Eastern studies on bodies and gender face the same issue regarding gender representation. Men's bodies have received less attention than women's bodies in academic research (Wolf, 2013; Faludi, 2009; Bordo, 1993). Therefore, achieving a balance between studies on men's and women's bodies can provide a clearer picture of the inequalities between men and women in terms of body representation and societal expectations.

### Most social studies in bodies focus on one race

The majority of academic research, and in particular, Western studies, has sought to study race and body by focusing more on white women and their concepts of beauty, self-esteem, body image, and body satisfaction, in comparison with other races such as Black, Latino, and others (Tiggemann, 2011; Jaffee and Mahle Lutter, 1995; Casanova, 2004). In addition, the intersection of race and gender has received increased attention in recent years (Casanova, 2004). Some studies have argued that race and gender are both structures of social differentiation “whose markers are borne on the body” (Morris, 2002: 133). Social scientists, psychologists, and scholars from other academic disciplines have sought to analyze how the Western ideal of body image can contribute to lower body image and self-esteem levels among non-white racial groups (Rucker III and Cash, 1992).

However, recent studies in this field have found that African American women tend to have higher levels of positive body image and self-esteem compared to white women, which is largely attributed to a rejection of white beauty standards (Rucker III and Cash, 1992; Jaffee and Mahle Lutter, 1995; Casanova, 2004).

In recent years, some studies have been done about body acceptance among different races such as African American, Asian American, white or non-white Hispanic/Latina, and multiracial groups. For example, some studies examined how race impacts body acceptance among majority-race women as compared to ethnic minority samples. Most of this research was conducted with white women and measured their level of body satisfaction compared to that of minorities (Grabe and Hyde, 2006; Hrabosky and Grilo, 2007; Kashubeck-West et al., 2013; Lovejoy, 2001; Schaefer et al., 2018). More studies also sought to analyze the body and body image and body acceptance by using a comparative framework of white European or American women (Smolak and Striegel-Moore, 2001). Many studies have found that white women worry more about their weight, have lower body satisfaction, more desire to hide body size with clothing, a higher level of eating disorders, and a higher level of body-related depression and anxiety compared with women from other racial groups (Rucker III and Cash, 1992; Roberts et al., 2006; Hesse-Biber et al., 2010). Research suggests that cultural and societal pressures to attain a thin ideal are more pronounced among white women, contributing to heightened body dissatisfaction and a greater prevalence of disordered eating behaviors (Grabe and Hyde, 2006; Shaw et al., 2004). These findings highlight the racial disparities in body image concerns and the influence of sociocultural factors on body perception and mental health.

Moreover, several studies have examined body image and dissatisfaction by comparing white and African American women. Strings (2019) emphasizes that fatphobia has historical racial origins, where Black bodies (African American women) have been systematically marginalized and excluded from Western beauty standards. Research suggests that, overall, Black women tend to report greater body satisfaction and lower levels of body dissatisfaction compared to their white counterparts (Overstreet et al., 2010; Merten et al., 2008; Roberts et al., 2006). In addition, Black women have fewer preoccupations with weight and have a lower level of eating disorders (Kronenfeld et al., 2010). Black women are also more satisfied with their bodies compared to other women of different ethnic and racial groups (Sanderson et al., 2013). Black women are happier with their bodies and they appreciate the special features of the race such as skin color, body shape, and lip size (Bond and Cash, 1992; Mucherah and Frazier, 2013). Furthermore, some researchers have attributed the differences in body image and satisfaction between African American and white women to the historical legacy of racism against African American, which has given them greater emotional resilience to overcome negative comments about their bodies from the dominant culture (Stombler and Padavic, 1997). Additionally, some scholars have found that African American strongly adhere to their cultural identity, which enhances their body appreciation in contrast to the body standards of the dominant race (Brook and Pahl, 2005).

Although most of the literature related to race, body, and gender has focused on themes such as beauty, self-esteem, body image, body satisfaction, and eating disorders, few studies have examined the impact of the dominant culture (e.g., body image and beauty standards of white women in Western culture) on minorities and how these influences affect the body image and body satisfaction of minority groups (Hesse-Biber et al., 2010). Additionally, little attention has been given to issues related to body and race, such as the experiences of minorities in different settings (e.g., college, school; Hesse-Biber et al., 2010). For example, some have studied the complexity of the relationship between the increased acculturation of dominant culture like the norms of White beauty and its impact on Black women's bodies (Roberts et al., 2006; Smolak and Striegel-Moore, 2001). They found that Black women who attend colleges where there is a predominance of white women have more eating disorders (Roberts et al., 2006; Smolak and Striegel-Moore, 2001), and are more concerned with body image than other Black women (Stice et al., 1994) and other women of color (Mulholland and Mintz, 2001; Williams, 1994).

In addition, much literature in recent years has shown that the standards of body image and satisfaction are different across cultures and historical periods, and that women experience their bodies in many different ways (Chrisler and Johnston-Robledo, 2018). For example, although Latino women and African American women are less disturbed with heavier bodies than White women, Asian women prefer to be extremely thin unlike White women (Guan et al., 2012). Moreover, studies have shown that young Korean women are more dissatisfied with their bodies than young American women (Choi and Choi, 2016). Some studies compared body dissatisfaction between Asian American females and other racial groups, and the result showed that Asian American females have greater body dissatisfaction than their European, Hispanic, and African American peers (Forbes and Frederick, 2008; Kennedy et al., 2004). They are also more concerned with body weight and eating disorders than White women (Nouri et al., 2011).

Most academic research and studies focused on body and race in different academic disciplines have examined how race can influence the body. Recently, more attention has been paid to the intersection of race and gender (Casanova, 2004) which can give a better understanding of how women/men of different races see their bodies in comparison to the dominant race. Most studies in different academic disciplines have sought to analyze the connections between race and body and how this relation can influence beauty, self-esteem, body image, body satisfaction, and body acceptance among races (Rucker III and Cash, 1992; Jaffee and Mahle Lutter, 1995; Casanova, 2004). These studies and scholar's contributions open doors for more studies and questions, such as, can race and body work differently in non-western societies, how can governments and official institutions play a role in reducing the influence of the perception of the body through race, social and economic status, as well as social life?

### Most social studies in bodies focus on western societies

Most social studies on the body focus on Western societies (e.g., the United States and Europe) while giving little attention to other parts of the world (e.g., Middle Eastern societies, China, India, etc.). This trend can be attributed to the increasing academic interest in studying the body and the social experience of embodiment, which emerged in the 1990s in most Western societies (Shilling, 2007; Turner, 1997). The body, as a topic in sociology, has become a focal point for understanding comprehensive social life in Western studies. Various phenomena, problems, and issues in society are connected to the human body, influencing both everyday life and bodily experiences. The increasing focus of studies on some issues related to the body in sociology, as well as the development of medicine, health, and illness in Western countries, this led to the founding of the Journal of Body and Society which was first published in 1995 (Boero and Mason, 2020). In addition, the American Sociological Association recognized a new area of study about the body and embodiment in 2008 (Boero and Mason, 2020). Despite the growing academic interest in body studies within Western societies, this focus has not been equally extended to the study of bodies in other parts of the world. There remains a significant gap in sociological research regarding non-Western perspectives on the body and embodiment.

Literature on bodies in the English language in other parts of the world is also rare (Jafar and de Casanova, 2013a). Studies in this field have focused almost exclusively on the United States and Europe while most people live outside the United States and Europe (Jafar and de Casanova, 2013b). Therefore, studies and findings (In English Language) on the body in non-Western parts of the world remain significantly fewer compared to those conducted in Europe and the United States. In addition, it is hard to cover all the world, but at least there needs to be an attempt to produce comparative studies about the body in other parts of the world with Western studies. Therefore, achieving balance in this field helps to develop comprehensive body studies in terms of the nature of the body in different cultures and how individuals in these different cultures interact with their bodies.

### Most social studies on the body do not focus on fatness as a social issue

Over the last few decades, the increase in gender studies has contributed to the emerging field of fatness studies (Harjunen, 2009). This field focuses on the social, cultural, psychological, economic, and historical aspects of fatness and seeks to explain how fat individuals are treated and represented. It views body weight and size as carrying a set of social meanings, which vary depending on gender, traditions, values, and cultural contexts. Consequently, these meanings may change over time and across different societies (Sobal and Maurer, 1999; Harjunen, 2009). Therefore, fatness is perceived differently between cultures and across history in terms of accepting the fat body or not based on the social discourse in society (Harjunen, 2009). Even though studies on fatness and its effects have increased over the last decade, further research is needed to explore more areas of social life. This remains a fertile and expansive field that allows researchers to investigate various social phenomena, particularly those related to fatness and gender.

Some scholars analyze theoretical approaches to the study of fatness, and they categorize the study of fatness and weight as objectivist or constructivist (Sobal and Maurer, 1999). The objectivist approach tends to describe the problems associated with fatness (obesity, ill health, etc.), and concentrates on describing the problem of fatness, while the constructivist approach concentrates on the ways an individual's body is defined based on its weight. It also focuses on the fact that, more than ever, a greater number of people are overweight (Sobal and Maurer, 1999; Harjunen, 2009).

A constructivist approach studies the processes whereby various social actors and institutions have erected boundaries between normative and non-normative bodies (Foucault, 1979). As a result of such processes, the bodies are seen as different and separated based on the judgement of society as to what determines. fatness or thinness, or healthy bodies and unhealthy bodies (Harjunen, 2009). All of these differences between bodies, either normative or non-normative, are considered social constructions. Thus, normative and non-normative bodies are linked to the social meanings that reflect the history, culture, tradition, values, and norms of certain nations. For example, social acceptability and respectability have always been linked to body size. Historically, the respectable body of both men and women [LA(1] was stout and sturdy, but now the respectable body is thin (Featherstone, 1982). This is especially so for women, and increasingly so for men as well (Galioto and Crowther, 2013; Cordes et al., 2017).

The gap in academic research regarding fatness and the body in feminist literature is evident. First, most research on fatness has been predominantly dominated by the medical paradigm (Harjunen, 2009). The focus has primarily been on viewing fatness as a medical issue. Medical analyses of fatness and the body have been widely disseminated, particularly in relation to specific medical concerns associated with fatness. For example, there were a large number of research projects about the ideal of thinness, dieting (e.g., Orbach, 1993; Bordo, 1993), body norms and ideals, eating disorders (e.g., MacSween and Macsween, 2013; Hesse-Biber, 1996), but fatness and the experiences relating to being fat did not get more attention, and some studies do not deal with fatness directly but emphasize thinness or dieting (Harjunen, 2009). In addition, the majority of studies about fatness came from medical and psychological studies about the dangers of obesity (Harjunen, 2009). As a result, there is a limited amount of social scientific research on fatness. However, scholars have emphasized the importance of viewing fatness as a social phenomenon rather than merely a medical issue (Pausé and Taylor, 2021). Fatness is a social phenomenon, and it is part of social reality that needs analysis and investigation by social science to understand its experience among different genders, races, and classes and how it impacts the everyday life of individuals (Pausé and Taylor, 2021). So, there is a limited amount of social scientific research on fatness. Fatness is a social phenomenon, and it is part of social reality that needs analysis and investigation by social science to understand its experience among different genders, races, and classes and how it impacts the everyday life of individuals.

### Social studies on bodies, gender, and fatness in the middle east: present and future

The importance of the body in sociological studies has increased for several reasons. First, the historical development of medicine, consumerism, and industry has contributed to understanding and analyzing the body as a system with meaning and a specific structure. In the 19th century, the rapid rise of consumerism and industrial development heightened interest in enhancing the body's efficiency and capabilities through exercise, diet, and management. These practices contributed to the regulation of the body and the promotion of better health (Shilling, 2012; Boero and Mason, 2020).

In the 20th century, the entertainment industry expanded significantly, and its development was remarkable. The greatest focus was then on industries that valued consumption more than production (Turner, 1992). According to some scholars, “The transition from the Renaissance to the modern world thus involves a transition from the ‘open body' linked to the public world through ritual and carnival to the ‘closed body' of individualized consumer society” (Turner, 2008, p. 39; Adelman and Ruggi, 2016). The body, especially the external body, was the cornerstone of this development. The body has become a central element in the commodification of everyday life. Advances in medical health and beauty have also influenced perspectives on treatments such as plastic surgery, which, in various ways, reshape the body to align with cultural expectations or evolving global standards, either directly or indirectly (Turner, 1992). In addition, the development of entertainment industries such as sports and film, along with cultural openness between countries, increased travel and cultural convergence, and the rapid advancement of media, has turned the body into a primary marketing tool. This has also led to the creation of body preferences within societies, such as the idealization of a thin and muscular body based on gender norms, as well as negative reactions toward physical forms that do not conform to these standards (e.g., a fat body; Turner, 1992).

On other hand, we can see the important development of tech and how this development of tech impact on our lives including our bodies (Arnaldi et al., 2024). For example, social media platforms such as Instagram, TikTok, Snapchat, Facebook, and X function as sites of body surveillance and self-regulation, where individuals construct and curate their digital selves based on dominant beauty ideals. Studies suggest that these platforms reinforce unrealistic beauty standards, influencing body image perceptions and self-esteem (Aparicio-Martinez et al., 2019; Perloff, 2014). Research also indicates that social media usage is associated with increased body dissatisfaction, particularly among young women and adolescents (Fardouly et al., 2015; Tiggemann and Slater, 2013). Furthermore, the algorithmic nature of these platforms amplifies content that idealizes certain body types, contributing to a culture of self-comparison and validation-seeking behavior (Holland and Tiggemann, 2016).

Given these dynamics, it is essential for body studies to integrate digital subjectivities as a key element in examining contemporary body politics. Future research should delve deeper into the intersection of technology, identity, and embodiment, recognizing influences of digital spaces in shaping body norms

Moreover, future studies should address several gaps in body research, such as: examining how different cultural contexts influence body image perceptions and body representations, especially in non-Western societies (e.g., the Middle East, Asia, and Africa). Investigating how shifting cultural norms and societal expectations affect body perceptions over time, particularly in relation to gender, race, and class. In addition, incorporating sociological, psychological, and anthropological perspectives to provide a more holistic understanding of body image formation across different cultural and historical contexts. Moreover, exploring how government regulations, public health initiatives, and socio-economic policies impact body norms and societal attitudes toward body diversity.

While there has been an increasing focus on body studies in various cultural contexts, research on the Middle East remains limited. Although this study touches upon the region, it does not extensively reference Middle Eastern scholars or in-depth research from the area. Future research should aim to incorporate contributions from Middle Eastern scholars to enrich the understanding of body norms beyond Western frameworks.

Feminist scholars such as Abu-Lughod (2015) and Mahmood (2013) provide critical tools for analyzing body politics in Middle Eastern societies. While this paper does not delve deeply into their frameworks, their work can serve as a foundation for developing alternative methodologies and epistemologies that challenge other perspectives, such as those critiqued by Donna Haraway.

In conclusion, in the last few years, scholars have increasingly paid attention to the body as an important aspect of individual identity. They have studied race, class, and gender and how they are related to the body. However, it is rare to find studies that emphasize the body and fatness across cultures. In other words, many researchers have studied and analyzed the body in Western societies, but we have few studies that have made cross-cultural analyses of the experiences of bodies (Gailey and Harjunen, 2019). In addition, many scholars have studied women's experiences of their bodies, but as of yet, not much scholarship has focused on men (Burlew and Shurts, 2013), even though it is a growing field (Daniel and Bridges, 2013; Castonguay et al., 2014).

## Methodology

### Research design

This study employs a systematic literature review to analyze the spectrum of coverage and focus distribution on body characteristics in existing literature. The purpose is to identify trends, gaps, and the extent of research on various body characteristics across different demographic variables.

### Literature search strategy

A comprehensive search was conducted in academic databases, including PubMed, PsycINFO, Scopus, Web of Science, and Google Scholar. The search terms used were “Body image,” “Body satisfaction,” “Body dissatisfaction,” “Race and body image,” “Gender and body image,” “Fatness and body image,” and “Body perception.”

### Inclusion and exclusion criteria

The study included peer-reviewed journal articles and scholarly books published between 1959 and 2024, covering body characteristics in relation to gender, race, socio-economic status, fatness, and cultural contexts. Both qualitative and quantitative research studies were incorporated. Given the foundational role of classical sociological theories (e.g., Goffman, 1959; Foucault, 1979; Bourdieu, 1984), older works were included alongside recent empirical studies (e.g., Pausé and Taylor, 2021; Arnaldi et al., 2024). Additionally, cross-cultural and interdisciplinary studies that examine body image across different societies (e.g., Gailey and Harjunen, 2019; Turner, 2008) were considered relevant.

### Exclusion criteria

The study excluded: non-peer-reviewed sources, such as blogs, news articles, and opinion pieces. Studies not available in English, to maintain consistency in analysis. Medical and psychological studies that focus solely on obesity as a health issue without addressing its social and cultural implications.

### Data extraction

An initial screening of titles and abstracts was conducted, followed by a full-text review of selected articles. A standardized data extraction form was used to collect relevant information, including author(s), year of publication, study objectives, sample characteristics, methodology, key findings, focus areas, and identified gaps.

### Analysis

Descriptive statistics were used to summarize the number and types of studies focusing on each body characteristic. Thematic analysis was conducted to identify common themes and trends. Findings were synthesized to highlight how different body characteristics are portrayed and studied across various demographics, and gaps in the literature were identified.

### Presentation of results

The results were presented in a descriptive summary, including tables and charts to visually represent the focus distribution. A thematic synthesis provided a detailed discussion of the main themes, insights into the portrayal of body characteristics across different populations, and highlighted areas with limited research.
